# Wastewater surveillance in Pakistan: Preventing future epidemics

**DOI:** 10.1016/j.amsu.2022.104495

**Published:** 2022-08-24

**Authors:** Wajeeha Bilal Marfani, Laiba Imran Vohra, Abdul Moiz Sahito, Abdullah Nadeem

**Affiliations:** Department of Medicine, Dow University of Health Sciences, Karachi, Pakistan; Department of Medicine, Ziauddin University, Karachi, Pakistan; Department of Medicine, Dow University of Health Sciences, Karachi, Pakistan

Correspondence,

The surveillance of wastewater is the process of monitoring contaminants in wastewater. Biosurveillance, detection of pathogens in local populations, and detection of psychoactive drugs are some of its uses. In Pakistan, the wastewater that comes out of households and industries goes either directly into a sewer system, a natural drain or water body, a nearby field, or an internal septic tank [1]. There is no biological treatment process in most cities, except for Islamabad and Karachi, which treat only a small proportion (8%) of their wastewater before disposal [1]. Therefore, wastewater monitoring and treatment are essential to ensuring the health of multiple ecosystems.

A majority of disease outbreaks that occur during the monsoons are water-related, significantly contaminated water. A lack of potable water during the monsoons, along with surface water contamination by flood or sewage, leads to most water-borne diseases. Due to the ravages of the monsoon on electricity supply, any electricity-driven water filtration system is rendered ineffective [2].

According to the WHO, water-related diseases kill more than 3.4 million people worldwide annually, making water contamination the world's leading cause of disease and death [2]. Children are the majority of victims, and they typically die from illnesses caused by organisms that thrive in water contaminated with raw sewage [2].

Although Pakistan does not have a dedicated study facility for wastewater, various studies have reported numerous enterotoxins in Pakistani wastewaters. According to the study by Tanzeel Zohra et al., the presence of *V. cholerae* in wastewater might be concerning for possible cholera outbreaks in the nation. According to their study, the proportion of *V. cholerae* distribution varied by Pakistani province, with Balochistan having the greatest distribution (20%), followed by Punjab (16%), KPK (15%), and Sindh (11%) [3]. Transmission of the SARS-CoV-2 virus may also be related to water, sewage, and the dangers posed by the wastewater industry during the COVID-19 epidemic. According to studies, the monitoring of wastewater could be a useful early-warning tool for the spread of COVID-19 and other health threats. It is believed that SARS-CoV-2 enters wastewater through the stool of infected individuals and can survive in stools for up to 4 days [4,5]. The study by Salman Sharif et al. used current polio surveillance channels to investigate the presence of SARS-CoV2 in wastewater. The analysis revealed that COVID-19 was present in the sewage from 13 districts. As the prevalence of COVID-19 changes over time in different communities, wastewater testing is a novel and unique approach for monitoring it [5].

Even though wastewater surveillance in Pakistan has matured into an effective strategy for various outbreaks, it often comes with challenges. The major issues that need to be considered are accounting for short-term fluctuations in population density for a reflection of the population, the necessity for biomarker extraction technology for the complicated wastewater mixture, and inadequate analytical tools that cover the susceptible areas [6]. Researchers aren't always familiar with how decisions are made in public health settings and have problems with data actionability and understanding [7]. Developing countries typically do an inconsiderable job of adequately treating fecal waste and wastewater, and in many situations, wastewater is simply released into lakes and rivers without any kind of treatment [8]. Because the majority of homes are not linked to sewerage networking, there are additional challenges with wastewater-based epidemiological tracking and monitoring of epidemics in the future [8]. Unfortunately, there is no unified system that generates statistics for the large number of inhabitants living in underdeveloped areas, making it unattainable to create and implement a legislative framework in developing nations like Pakistan [9].

The procedure of testing wastewater for pathogens is best discussed using SARS-CoV-2 as an example. People infected with SARS-CoV-2 can excrete viral RNA (viral genetic material), and this RNA can be found in communal wastewater [10]. As it flows into a treatment facility, wastewater from a sewershed (the community area served by a wastewater collecting system) is collected. SARS-CoV-2 testing is performed on the samples at environmental or public health laboratories. The presence of SARS-CoV-2 in wastewater is detected in patients with and without symptoms. It is possible to detect infection in a community and determine if levels are increasing or decreasing by monitoring concentrations of SARS-CoV-2 in wastewater using genomic sequencing [10]. The Pakistan Council of Research in Water Resources (PCRWR) assesses the effects of domestic and industrial effluents in order to determine wastewater treatment needs [11].

Local governments in all nations are in charge of the actual implementation and operation of wastewater treatment and monitoring facilities. Academic/research organizations and the corporate sector are primarily responsible for sample analysis and the creation of technical solutions, including monitoring equipment [12]. PCRWR's microbiological testing kit and arsenic testing kit for pathogenic and chemical surveillance are examples of such equipment [11]. Numerous government agencies, like the Pakistan Council of Research of Water Resources and Pakistan Environmental Protection Agency, are actively engaged in observing the effects of wastewater application on agricultural fields. These organizations promote research with financial and technical assistance in addition to studying the long-term effects of wastewater on soil characteristics.

As a result, while developing a national platform for wastewater surveillance systems, it is necessary to include all authorities [12]. The government should ensure that the governance structure for wastewater surveillance systems is not only a stopgap solution, but rather serves as an early warning system for future pandemics. Virome analysis of wastewater, as demonstrated during the COVID-19 pandemic, could discover new viruses before they are clinically recognized in a population [13]. Public health experts are working on a pilot study in which sewage water in Karachi will be analyzed for the presence and concentration of coronavirus. The World Health Organization's Pakistan office, the National Institute of Health (NIH), and infectious disease epidemiologists from Agha Khan University (AKU) will work together on the project to demonstrate that such measures are achievable in a nation like Pakistan [14].

There are several methods for monitoring wastewater. The two techniques that are most often used are community wastewater monitoring and targeted wastewater monitoring. The former technique uses pathogen-sampling wastewater as it enters a treatment facility (known as untreated influent) to examine infection patterns among the population that supplies water to the sewer system. The number of treatment facilities for community-level wastewater monitoring is determined by the region's need for public health data and the availability of resources [15]. On the other hand, targeted wastewater monitoring requires sampling wastewater from upstream in the wastewater network (e.g., lift stations, interceptors, manholes). Targeted wastewater surveillance may help researchers better understand how some infections spread inside a sewershed [16].

In order to provide our health authorities with more information on early indications of enteroviral infections, routine monitoring of Pakistan's water and wastewater quality should be conducted in view of the increased transmission of viral and bacterial enterotoxins. If we are aware of the habitats of these organisms and how they are spread, we will be able to fight against these illnesses before they may cause disease.

In conclusion, wastewater surveillance can be used in conjunction with clinical surveillance to identify possible transmission indicators, facilitating more proactive public health interventions. Communication between laboratories, researchers, companies, and public health regulators, is necessary for sustainable wastewater surveillance. Long-term gains from investments in wastewater surveillance systems can act as a non-invasive early alert system for outbreaks and help governments improve their ability to respond to public health emergencies quickly and accurately. Further studies are required to evaluate the utility of wastewater surveillance to direct policy and public health interventions.

## Ethical approval

N/A.

## Sources of funding

None.

## Author contribution

All authors equally contributed.

## Registration of research studies

1. Name of the registry: N/A.

2. Unique Identifying number or registration ID: N/A.

3. Hyperlink to your specific registration (must be publicly accessible and will be checked): N/A.

## Guarantor

N/A.

## Consent

N/A.

## Declaration of competing interest

None declared.
